# The impact of the COVID-19 pandemic on healthcare access and utilisation in South Sudan: a cross-sectional mixed methods study

**DOI:** 10.1186/s12913-022-08929-9

**Published:** 2022-12-20

**Authors:** M. A. S. Schots, H. L. S. Coleman, G. W. Lutwama, M. Straetemans, E. Jacobs

**Affiliations:** 1 KIT Royal Tropical Institute, Mauritskade 64, Amsterdam, 1092 AD The Netherlands; 2 Health Pooled Fund, American Embassy Residency Road, Juba, South Sudan

**Keywords:** Healthcare access and utilisation, COVID-19, Fragile- and conflict affected settings, South Sudan

## Abstract

**Background:**

Indirect effects of the COVID-19 pandemic on communities in fragile and conflict-affected settings may be severe due to reduced access and use of healthcare, as happened during the 2015 Ebola Virus Disease outbreak. Achieving a balance between short-term emergency response and addressing long-term health needs is particularly challenging in fragile and conflict-affected settings such as South Sudan, given the already significant barriers to accessing healthcare for the population. This study sought to characterise the effect of COVID-19 on healthcare access and South Sudan’s healthcare response. This can inform efforts to mitigate the potential impacts of COVID-19 or other epidemiological threats, and contribute to understanding how these may be balanced for greater health system resilience in fragile contexts.

**Methods:**

We conducted a mixed methods study in three of South Sudan’s states, combining data from a cross-sectional quantitative household survey with qualitative interviews and Focus Group Discussions.

**Results:**

Even though some fears related to COVID-19 were reported, we found these did not greatly dissuade people from seeking care and do not yield significant consequences for health system programming in South Sudan. The pillars of the response focused on risk communication and community engagement were effective in reaching communities through different channels. Respondents and participants reported behaviour changes that were in line with public health advice. We also found that the implementation of COVID-19 response activities sometimes created frictions between the national government and international health actors, and that COVID-19 caused a greater reliance on, and increased responsibility for, international donors for health planning.

**Conclusions:**

Given the fact that global priorities on COVID-19 are greatly shifting, power dynamics between international health agencies and the national government may be useful to consider in further COVID-19 planning, particularly for the vaccine roll-out. South Sudan must now navigate a period of transition where COVID-19 vaccine roll-out continues and other domestic health burdens are re-prioritised.

**Supplementary Information:**

The online version contains supplementary material available at 10.1186/s12913-022-08929-9.

## Background

From the start of the COVID-19 pandemic, early projections suggested the indirect health effects of the pandemic could be severe: unless countries minimised the disruption of key health services due to COVID-19, secondary mortality could outweigh the already-significant death toll from the Coronavirus itself [1–3]. This was a direct lesson from mortality trends observed during the 2014–2015 Ebola Virus Disease (EVD) outbreak in Western Africa [1, 4] where the redirection of health system efforts towards EVD resulted in substantial additional barriers to accessing care, causing an estimated additional 10,600 deaths from just malaria, HIV/AIDS and tuberculosis, which almost equals the estimated 11,300 deaths caused by EVD directly [4–6].

As with EVD, it continues to be vital for countries to balance their pandemic response with the continued delivery of routine health services [1, 2]. Achieving a balance between short-term emergency response and addressing long-term health needs is particularly challenging in fragile and conflict-affected settings such as South Sudan, given the already significant barriers to accessing healthcare for the population [7]. A long history of civil conflict, natural disasters and underinvestment in basic services has created the circumstances where over two-thirds of the population are in need of humanitarian assistance [8]. Approximately, just 44% of the South Sudanese population lives within 5 km of a health facility and has consistent access to primary care services [9]. Addressing the significant capacity and resource constraints of the healthcare system, linked with the politically-fragile situation in South Sudan, is the responsibility of a combination of domestic and international actors and organisations [4, 7, 10]. Given the combination of limited access to care, the involvement of these various actors, and the insecure political and resourcing environment, the South Sudanese health system is especially vulnerable to external pressures and health shocks, such as COVID-19 [11].

In 2020, initial epidemiological projections of COVID-19 painted a grim picture of the potential impacts on health outcomes due to COVID-19-induced healthcare disruptions, due to reduced access and utilisation of essential health services by the population [12, 13]. Even though major variations have been reported in terms of COVID-19's impact on healthcare access in sub-Saharan Africa, confounded with the impact of lockdown measures [14–16], a recent analysis of COVID-19’s impact on service utilisation rates in the Democratic Republic of the Congo (DRC) [17] found an immediate decrease in the utilisation of health services following the onset of the COVID-19 pandemic.

While studies of COVID-19’s indirect impact on health are ongoing in countries across the world [18, 19], no studies have been conducted thus far on how healthcare access and utilisation have changed in South Sudan due to COVID-19. Further, little is documented about how domestic health systems and international health actors can effectively respond to the COVID-19 and future pandemics, particularly in fragile and conflict-affected settings. An effective, coherent response in such settings is complicated by policy fragmentation, weak institutional capacity and the competing priorities of, and power dynamics between, domestic decision makers and international humanitarian and development actors. Insight into the nature and extent of COVID-19’s impact on healthcare access and utilisation in South Sudan can inform such a response to mitigate its potential impact, or the impact of other epidemiological threats, with a view to strengthening the resilience of health systems in fragile contexts. The aim of this study was therefore to understand the effect of COVID-19 on healthcare access and South Sudan’s healthcare response, in order to inform health planners and decision makers.

## Methods

We conducted a mixed methods study in three of South Sudan’s ten states, using an exploratory case study design, drawing from a cross-sectional quantitative household survey as well as qualitative interviews and Focus Group Discussions (FGDs). The mixed methods study design was used to provide a holistic understanding of health service utilisation, perspectives on healthcare services, and health seeking behaviour [20, 21]. The study was carried out iteratively in two phases: a qualitative phase followed by a quantitative phase. This sequencing allowed us to incorporate findings from the qualitative phase into the study methodology and survey questionnaire for the quantitative phase, helping us focus on certain themes over others. The central research question guiding this study was: ‘How has South Sudan’s response to COVID-19 matched its direct and indirect impacts on healthcare access and utilisation?’. The study was embedded within a larger study into healthcare access and utilisation in South Sudan, conducted by KIT Royal Tropical Institute (KIT) as the operational research partner for the Health Pooled Fund in 2020–2021. In response to the emerging COVID-19 pandemic and imported COVID-19 cases into South Sudan, preliminary questions on COVID-19 and its effect on healthcare access were integrated into the household survey questionnaire and qualitative research tools.

Following our research question, our methods can be categorised into two parts. In the first part, we focused on the COVID-19 situation in South Sudan and the response of governmental and non-governmental health actors to the pandemic. In the second part, we focused on the impact of COVID-19 on healthcare access and utilisation in South Sudan.

To document how the COVID-19 situation in South Sudan evolved over time, we conducted a literature review of both scientific and grey literature, particularly policy reports and newspaper articles [22]. To compare healthcare access and utilisation across states, we used data from a household survey, Key Informant Interviews (KIIs), semi-structured interviews and FGDs [Table 1]. Additionally, we conducted four follow-up (expert) interviews at the central level with Key Informants involved in health system management to substantiate or qualify findings about COVID-19's impact from the aforementioned data sources.

**Table 1:** Methodology, themes explored and participants/respondents per method

Method	South Sudan				
	Central Equatoria	Western Equatoria	Warrap	Central level	Total
Literature review^1,2^					
Key Informant Interviews^1,3,5^	2	2	2	4	10
Semi-structured interviews^3,4,5^	21	21	21	-	63
Focus Group Discussions^3,4,5^	9	9	9	-	27
Household Survey^3,4,5^	224	709	290	-	1,223

Number of FGDs does not correspond to the total number of participants and for the household survey, these numbers represent the numbers of households surveyed (not individuals). All data was collected between August 2020 and July 2021. Themes explored:

^1^South Sudan’s COVID-19 response

^2^Development of COVID-19 in South Sudan

^3^Community knowledge & awareness of COVID-19

^4^Perceived impact of COVID-19 on communities’ livelihoods

^5^Community response to COVID-19

### Study context

Due to high levels of institutional and social fragility as well as ongoing conflict in South Sudan, the World Bank Group has classified the country as a fragile state since the country gained independence in 2011 [23, 24]. Chronic conflict has left the health system underdeveloped and an estimated 70% of health services are provided by Non-Governmental Organisations (NGOs) and faith-based organisations [25]. South Sudan’s poor health outcomes, such as the world’s highest maternal mortality rate [26], are ascribed to low access to healthcare services, particularly for women, new-borns and children [23]. Furthermore, service coverage varies considerably at the county and state level [27].

The﻿ Health Pooled Fund (HPF) is a multi-donor funding mechanism that supports the Ministry of Health in providing basic primary healthcare services in eight of South Sudan’s ten states. HPF was launched in 2012 and was in its third phase (HPF3) in 2022. Funding comes from several donors^1^ and is delivered through HPF to eleven implementing NGOs. At the time of this study, the programme supported a total of 794 health facilities at different levels of care including 25 hospitals, 192 Primary Health Care Centres, and 577 Primary Health Care Units. HPF also supports the government-led Boma Health Initiative (BHI) which aims to increase demand, access and awareness of health services by strengthening community health systems.

### Selection of study sites

**Fig. 1:**
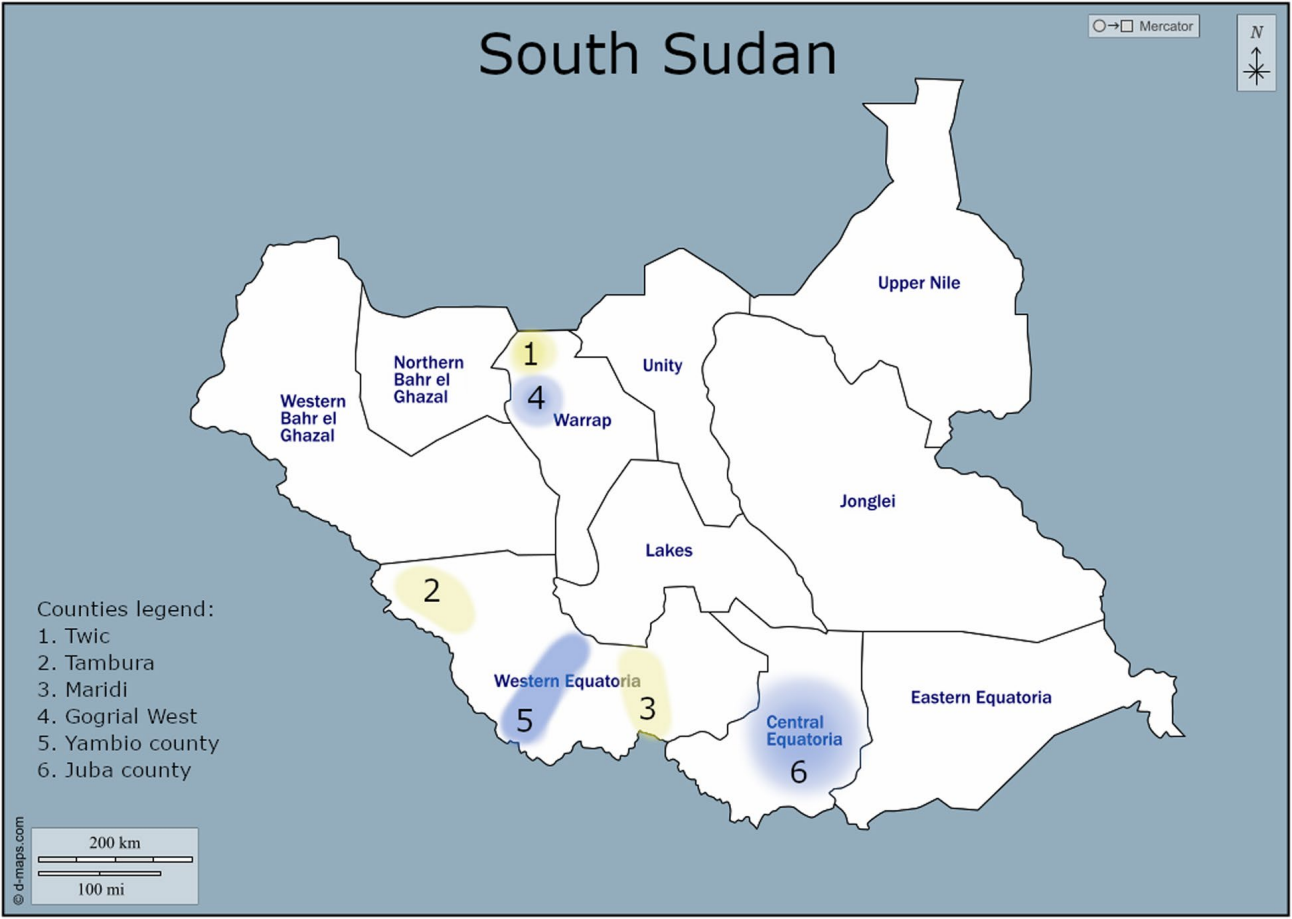
Map of South Sudan, illustrating the different sites of data collection. Interviews and FGDs were conducted in the counties highlighted blue (Juba, Yambio and Gogrial). Household survey data was collected in these same counties, as well as in Twic, Tambura and Maridi (highlighted yellow on the map)

For both the qualitative and quantitative arms of the study, we purposefully selected three states as study sites taking into account several selection criteria, such as accessibility, security, the availability of both urban and rural areas and the demographic characteristics of the population. Within each state, we purposively selected safe counties to achieve a diversity of the same selection criteria used at the state level. Central Equatoria, specifically Juba County, was chosen to represent an urban setting, whereas Western Equatoria and Warrap were selected to reflect a rural setting and rural-pastoralist setting, respectively. Figure 1 shows the different data collection sites.

### Sampling strategy & participants

#### Quantitative arm

The estimated sample size was based on the primary outcome measure: the proportion of the population who reported to have sought care, among those who needed care either due to sickness or disability or for other reasons, in the three months prior to the survey. The sample size was calculated using the single proportion formula with a 95% confidence interval, 5% margin of error and anticipating that 50% of those who needed care in the three months prior to the survey sought care [28].

We drew the sample using a multi-stage, cluster sampling design with random, systematic and purposeful selection of sampling units. In South Sudan, payams are the second-lowest administrative division, one level down from counties, and are further broken down into Bomas. For each state, the sampling frame consisted of safe, accessible and HPF-supported payams within the purposefully-selected counties and states. The primary sampling unit (PSU) was settlements. The 18 settlements were randomly selected from the list of eligible payams proportionate to their population size and were checked for habitation using satellite images. Following this, 21 households per settlement were randomly selected after all households in a settlement had been enumerated. The last sampling stage happened during data collection itself, where, in large households with more than ten household members who had needed care in the previous three months (i.e., all of whom would be eligible to complete the survey), the Open Data Kit (ODK) Collect survey application randomly chose ten household members to complete the remainder of the survey. This ten-person cut off was used to limit the time needed to complete the survey questionnaire for households with many members.

#### Qualitative arm

We conducted interviews with individuals involved in the implementation or management of healthcare services (e.g., HPF state coordinators and county health department officials) and with users and non-users of healthcare services (e.g., adolescent and adult community members); as well as FGDs with key stakeholders (e.g., Boma Health Committee members) and community members (e.g., persons with disabilities). KIIs were conducted with individuals who have a deep understanding of the health system at the central, state and county levels, to develop a rich description of the study topics. For the remaining participants, semi-structured interviews were chosen as they allow focused, conversational, and two-way communication. Additionally, FGDs were conducted with six to eight participants who share similar characteristics.

Participants were purposively selected through the HPF network. At the central level, four KII participants were selected based on them working for institutions involved in the health sector programming in South Sudan, at national or international level. All other participants were selected through snowball sampling starting from HPF state coordinators, state health officials and representatives of HPF-implementing NGOs and other non-HPF NGOs.

### Data collection

The interview and FGD topic guides as well as the household survey questionnaire were based on the healthcare access and utilisation framework of Levesque et al. [29] including aspects of both demand- and supply-side characteristics of access and utilisation.

Data was collected between August 2020 and June 2021 by the Forcier Consulting field team. In total, 10 KIIs, 63 semi-structured interviews, 27 FGDs and 1,223 household surveys were conducted. Interviews and FGDs were conducted by three trained researchers (one per state) who were selected based on their qualitative research experience, their ability to speak the state’s predominant language as well as English, and their knowledge of the state context. Translations of concepts in the topic guides from English to the language used in each state were agreed upon during trainings. Audio files of completed interviews and FGDs were downloaded to password-protected computers and transcribed. Audio files were directly translated into English, capturing as much of the original content as possible. Transcripts were reviewed for accuracy and completeness. The household survey was administered by trained enumerators. One survey was completed per household, to be answered by the heads of household or main care giver, answering on behalf of all household members. Forcier Consulting programmed and conducted the survey in Open Data Kit (ODK) on the SurveyCTO platform.

To contextualise findings from the household survey, FGDs and interviews, a purposive document review was conducted from February to June 2021. Documents were collected through scientific and general search engines. Searches were limited to documents published in the English language and peer-reviewed scientific literature as well as grey literature. The latter included mainly newspaper articles, as well as several policy documents such as South Sudan’s National COVID-19 Response Plan [30]. Abstracts and conclusions were read and, if information relevant to understanding South Sudan’s COVID-19 response was found, the document was included in the study. From reviewing these documents, we developed a narrative of South Sudan’s COVID-19 response. This narrative focuses on the time period up to the end of qualitative and quantitative data collection in July 2021 to contextualise the qualitative and survey responses describing this period of time. All epidemiological data included in this study is also limited to this time range.

### Data analysis

Following a qualitative content analysis approach, all interview and FGD transcripts were read repeatedly, and coded deductively based on the healthcare access and utilisation framework by Levesque et al. [29], after which sub-codes were created inductively. This led to a coding tree, which guided data analysis using Atlas.ti v8.4.25. Household survey data was analysed using Stata v15.1 SE. Descriptive statistics were calculated to understand the demographic and socioeconomic composition of the study population, as well as on variables of interest related to health seeking behaviour and COVID-19. These included variables at both the household and individual levels. Descriptive statistics were initially disaggregated by gender of the respondent and head of household, but the limited differences in responses by gender means the results presented in this paper are disaggregated by state only. We made a distinction between three distinct types of COVID-19 effects: direct health effects (infection with the virus), indirect health effects (effects of COVID-19 on communities’ ability to access and utilise care), and indirect non-health effects (e.g., school closures). Survey weights were applied when calculating frequencies to account for the sampling design.

To validate the findings, a research validation workshop was held with researchers involved in the HPF3 project. The workshop aimed to synthesise the study findings, by discussing main themes emerging from data analysis and sharing ideas on the most pressing issues with regards to healthcare access and utilisation in South Sudan during the COVID-19 pandemic. The validation workshop also allowed the researchers to contextualise and better interpret the findings in light of recent developments in the country.

### Ethical considerations

The protocol for the wider study into healthcare access was reviewed and approved by the ethics committee of both KIT and the Ministry of Health South Sudan. All research procedures were in accordance with the Declaration of Helsinki. Data collection adhered to KIT’s ethical standards with regards to neutrality, participation, informed consent, privacy and gender considerations. Respondents invited to the interviews, FGDs and household survey were informed about the intended (secondary) use of their data for dissemination and development purposes, and all participating individuals consented to this.

Interviews and surveys were conducted in a safe environment allowing for privacy and confidentiality. Only the research team had access to the data and identifiers were removed from transcripts. Prior to data collection, the research team was trained on research ethics to ensure guidance on ethical conduct was clearly understood and implemented. Confidentiality of the data collected was ensured by the means of password-protected storage.

## Results

### Part 1—COVID-19 in South Sudan: governmental structure and response

In March 2020, before South Sudan confirmed its first COVID-19 case (on April 5, 2020), a High-Level Task Force, including members from various ministries and civil organisations, was established to coordinate and communicate the measures needed to limit viral transmission in South Sudan [31, 32]. The task force would be advised by the COVID-19 National Steering Committee, a larger body consisting of a variety of governmental and non-governmental health actors [33]. Even though the steering committee would be advisory in nature, it was reported that donors and international actors were able to push the COVID-19 response considerably towards their own priorities and targets. In some cases, this resulted in greater attention towards certain pillars of the response, namely the supply of inputs like the procurement of protective equipment, whereas those pillars of the response focusing on managing health services and longer-term health system development remained under-resourced.

“The national government should be the one making the decisions, but they don’t entirely make them. They’re made depending on what the partners kind of push towards, or what the partners are adhering towards.” – Health Systems Technical Manager, donor

Both the High-Level Task Force and the National Steering Committee were guided by the Country Preparedness and Response Plan (later: National COVID-19 Response Plan) [30]. Measures to curb the spread of COVID-19 were implemented in March 2020 [33], through a two-pronged approach, focusing on the reduction of social interactions as well as limiting cross-border transmission of COVID-19 [Table 2] [31, 33]. In response to South Sudan’s first confirmed case on April 5, 2020 [33], the national government reinforced earlier lockdown measures and urged the public to strictly adhere to the social distancing guidelines [34]. Implementation of COVID-19 response activities was sometimes observed to create frictions between the national government and international health actors, for instance on responsibilities and remit. The Ministry of Health and national government were deemed to be responsible for coordinating the COVID-19 response, yet participants from international health agencies felt a lack of ownership and responsibility from the national government for the response.

**Table 2:** Measures implemented in South Sudan to curb the spread of COVID-19 [3, 34–37]

**Lockdown measures implemented in South Sudan during study period**	
24 March 2020	Initial lockdown measures for 30 days, including a ban on international and domestic commercial flights, the closure of border crossings, a ban on political, social and cultural gatherings (including but not limited to bars, night clubs and restaurants) and a nation-wide curfew
4 April 2020	First COVID-19 case confirmed in South Sudan
5 April 2020	Reinforcement of previous lockdown measures and social distancing guidelines
7 May 2020	Lockdown measures were partially lifted, schools remain closed
3 Feb 2021	Government of South Sudan announces a new nation-wide lockdown banning social gatherings, classes, religious services and political rallies following a sharp rise in coronavirus cases
29 Mar 2021	Number of weekly reported COVID-19 cases drops from > 1000 per week in February to less than 200 per week in March
4 May 2021	Lockdown lifted; schools reopened after over 14 months of closure
May – July 2021	Number of weekly reported COVID-19 cases remains below 100, no COVID-19 restrictions in place anymore

**Fig. 2:**
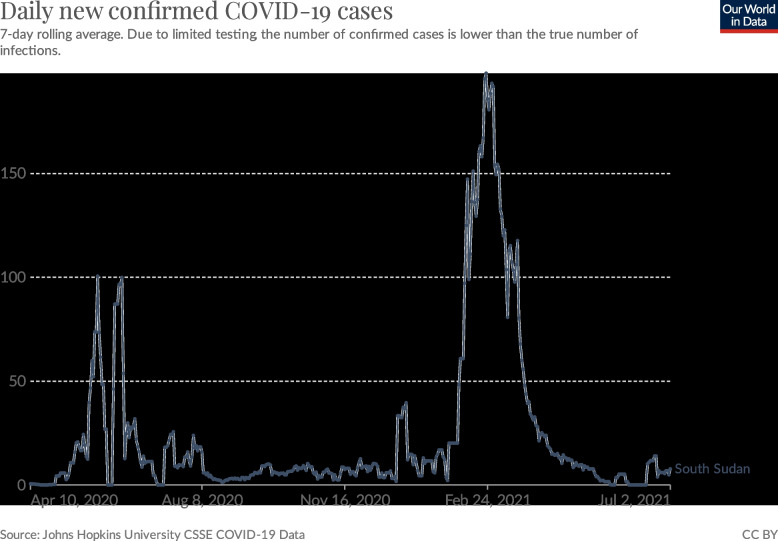
Daily new confirmed COVID-19 cases in South Sudan until July 1, 2021 (from: Our World in Data)

During the first two months of the COVID-19 outbreak in South Sudan, 1,317 official infections were recorded, mostly public officials in Juba, and 14 confirmed COVID-19 deaths [38]. Even though increasing numbers of confirmed COVID-19 cases were reported, the government decided to relax restriction measures on May 7^th^, 2020; a decision which was criticised in local news for being ill-timed and dangerous [35]. Following these relaxation measures, the number of confirmed cases doubled in May, from 111 new confirmed COVID-19 cases per week on May 11 to 332 new confirmed cases on May 18, 2020. Nonetheless, the effects of the COVID-19 pandemic in South Sudan appeared to remain rather moderate, relative to other countries, with a maximum of 481 new confirmed cases per week by the end of May [Fig. 2] [3].

Despite an increase in testing capacity in the beginning of June 2020, testing remained confined to particular areas of the country. Even though Juba-centred testing capacity was rolled-out to the rest of the country, by distributing Gene Xpert machines to laboratories in some states, interview participants suggested that reagents and skilled laboratory staff were often lacking which resulted in the machines not being used to their full capacity. Indeed, an analysis of laboratory data by the Sudd Institute suggested this may have hidden the true magnitude of COVID-19 transmission in South Sudan [39]. Furthermore, the same analysis reported the vast majority of the samples (97.7%) tested at South Sudan’s National Public Health Laboratory between April 2 and May 20, 2020 came from Central and Eastern Equatoria states, and predominantly Juba (Central Equatoria), the nation’s capital, and Nimule (Eastern Equatoria), a border crossing between South Sudan and Uganda. Despite the potentially hidden magnitude of COVID-19 transmission in South Sudan, participants from international health agencies felt that national leadership sometimes disproportionately focused on COVID-19, represented by the size of leadership bodies in charge of the response, and in contrast to the limited number of public health officials at the national and sub-national levels.

“At the national level of course, we [the national steering committee] have everybody from the donors, to the implementing partners to the fund managers, to just technical people, and then all kinds of random organisations. So, the national steering committee has I think [a] membership of over 200 at any given point” – Health Systems Technical Manager, donor

The combination of limited surveillance of COVID-19 transmission, due to weak testing capacity, and human resourcing—i.e., public health officials who would normally be in charge of overseeing the overall coordination of the health sector, redirecting their efforts towards the COVID-19 response—has had severe implications for the COVID-19 response.

**Fig. 3:**
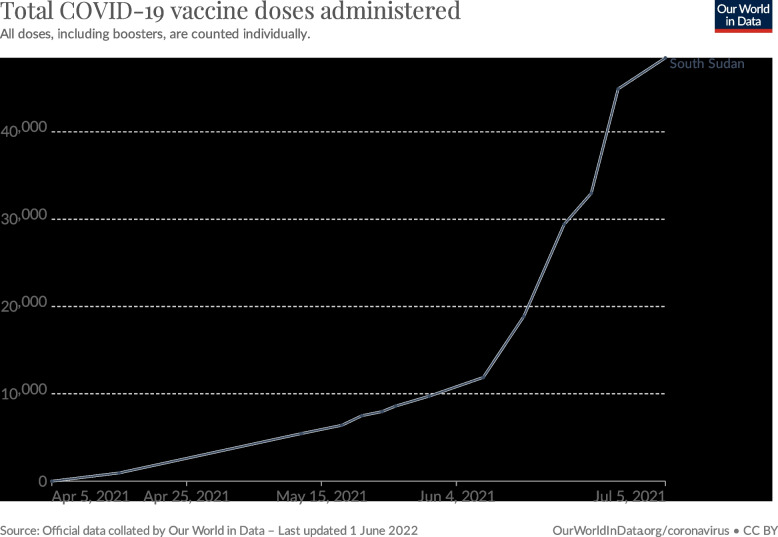
COVID-19 vaccine doses administered in South Sudan until July 5, 2021 (from: Ourworldindata)

In March 2021, South Sudan received 132,000 doses of the AstraZeneca vaccine through the COVAX Facility [40]. The vaccination campaign kicked off on April 6, 2021 and, over the following months, all frontline health workers were offered the vaccine, followed by people with known co-morbidities, including cardiovascular diseases and diabetes among others, and then people above 65 years of age [Fig. 3] [40, 41]. Following this cohort, vaccination progress slowed due to operational difficulties and low uptake. Consequently, South Sudan decided to return over half of its vaccines to the COVAX Facility as the doses would otherwise expire, and vaccination centres closed in mid-July 2021 [42].

#### COVID-19 Situation mid 2022

After data collection for this study ended, a third wave in COVID-19 cases was reported by the WHO in December 2021 [3]. After July 2021, more consignments of COVID-19 vaccines arrived in South Sudan and vaccine uptake improved. Nevertheless, a shortage of vaccine doses and logistics remained a continuous challenge in South Sudan. As of May 30, 2022, a total of 717,964 vaccine doses have been administered in South Sudan and 7.8% (625,723 people) of the target population (of 8 million) is fully vaccinated, either with two AstraZeneca doses or one Johnson & Johnson dose [43]. From the onset of the pandemic until March 2021, South Sudan has conducted approximately 340,000 COVID-19 tests [44].

### Part 2: Communities’ knowledge, attitude and perceived effects of COVID-19

#### Community knowledge and awareness of COVID-19

Based on the answers given by community members in the interviews and FGDs, almost all participants have heard of COVID-19 through different channels in the states surveyed: the symptoms were generally well-recognised, and participants were generally aware of the preventative measures. Even though two participants working in the health sector mentioned that misinformation around COVID-19 was circulating among communities, such information was not reported by community members themselves. Some community members did indicate gaps in their knowledge about COVID-19, for example on how to prevent transmission of the virus, and participants working in the health sector ascribed these gaps to incorrect examples set by community health workers, inefficient COVID-19 messaging or urban–rural differences in awareness campaigns. Nevertheless, most participants working in the South Sudanese health sector perceived the level of community awareness to be appropriate, which aligns to the answers given by community members.

#### Perceived impact of COVID-19 on communities’ livelihoods

Both the interview and household survey data suggested negligible direct health effects of COVID-19 in the three states surveyed. No interview participants reported having been infected or to personally know someone who had been infected. In the household survey, a negligible proportion (less than 1% across all three states) of respondents seeking care for a perceived need mentioned to have done so to get tested for coronavirus [Table 3].

**Table 3:** Household- and individual level findings related to COVID-19 and health seeking in South Sudan

Indicator	Central Equatoria			Warrap			Western Equatoria			Total		
	**N**	**%**	**SE**	**N**	**%**	**SE**	**N**	**%**	**SE**	**N**	**%**	**SE**
**Individuals total**	**1,744**			**4,756**			**2,116**					
**Individuals with a perceived need for care**	**767**	**49.7**	**1.8**	**996**	**52.6**	**1.8**	**2,142**	**47.8**	**1.9**	**3,905**	**49.3**	**1.1**
**Individuals seeking care for a perceived need**	**622**	**81.2**	**2.8**	**684**	**68.7**	**3.7**	**2,092**	**97.7**	**1.0**	**3,398**	**87**	**1.2**
**Individuals seeking care at a formal facility**	**590**	**95.5**	**1.2**	**633**	**93.2**	**1.9**	**2,062**	**98.6**	**0.7**	**3,286**	**97**	**0.6**
*Reasons for seeking care*												
COVID-19 test	**0**	**0**	**0.0**	**2**	**0.3**	**0.2**	**7**	**0.4**	**0.3**	**9**	**0.3**	**0.2**
Not related to COVID-19	**588**	**99.4**	**6.6**	**615**	**96.8**	**5.2**	**2,055**	**99.6**	**11.7**	**3,256**	**99**	**7.7**
Don’t know	**3**	**0.6**	**0.3**	**14**	**1.7**	**0.5**	**0**	**0**	**0.0**	**17**	**0.7**	**0.2**
No answer	**0**	**0**	**0.0**	**4**	**2.2**	**0.8**	**0**	**0**	**0.0**	**4**	**0.5**	**0.2**
**Individuals not seeking care for a perceived need**	**144**	**18.8**	**2.8**	**308**	**31**	**3.6**	**50**	**2.3**	**1.0**	**503**	**12.9**	**1.2**
*Reasons for not seeking care*												
Related to COVID-19^a^	**0**	**0.3**	**0.3**	**0**	**0**	**0.0**	**0**	**0**	**0.0**	**0**	**0.1**	**0.1**
Not related to COVID-19^b^	**157**	**98.7**	**25.0**	**330**	**97.4**	**18.2**	**59**	**97.8**	**57.7**	**550**	**97.8**	**18.5**
Don’t know	**2**	**1.1**	**0.6**	**8**	**2.3**	**0.9**	**1**	**2**	**2.1**	**11**	**1.9**	**0.6**
No answer	**0**	**0**	**0.0**	**1**	**0.3**	**0.2**	**0**	**0**	**0.0**	**1**	**0.2**	**0.1**
**Households total**	**224**			**290**			**709**			**1,223**		
**Households reporting indirect effect of COVID-19**	**201**	**89.6**	**1.7**	**62**	**21.4**	**3.4**	**701**	**98.9**	**0.4**	**964**	**78.8**	**1.8**
**Indirect effect of COVID-19 on health seeking**	**9**	**4.5**		**18**	**29.0**		**158**	**22.5**		**184**	**19.1**	
*Indirect effect of COVID-19*												
Mistaken similar symptoms for COVID-19	**3**	**1.7**	**0.9**	**9**	**13.8**	**6.9**	**81**	**11.5**	**2.0**	**92**	**9.6**	**1.5**
Lack/cancellation of health services	**3**	**1.6**	**0.8**	**5**	**7.9**	**3.4**	**31**	**4.4**	**0.7**	**39**	**4**	**0.6**
Other health problems due to not visiting a health provider	**3**	**1.6**	**0.8**	**4**	**6.7**	**3.2**	**46**	**6.5**	**1.7**	**53**	**5.5**	**1.2**

Survey weights are used in all calculations. N = number of individuals or households. *SE* Standard error

^a^Includes: fear of infections / gatherings, fear of being tested and fear of stigma

^b^Includes but not limited to: unavailability of drugs, no mode of transportation, poor quality of health services

In contrast to the limited direct health effects, non-health effects of COVID-19 were reported throughout the interviews and household survey. In the interviews, participants mentioned COVID-19 led to a lack of education due to school closures, teenage pregnancies, a lack of economic activities and church closures. Similarly, a considerable proportion of households (79%, *N* = 1,223), particularly those in the highest wealth indices [Supplementary Table 1, Additional File 1], perceived COVID-19 had affected their livelihoods in other ways than getting infected [Table 3], most notably through the disruptions to education, loss of income and separation from family members.

Indirect health effects of COVID-19 were also reported in the interview and household survey data. Some interview participants working in the South Sudanese health sector reported COVID-19 resulted in the reduced ability to access health services for the populations they served, due to reduced opening hours of health facilities, reduced availability of essential medicines and equipment, or reduced quality of care at facilities. Further, some interview participants stressed the reduced ability to seek healthcare for persons living with disabilities.

“Coronavirus came with the division for us in the hospitals, especially for us the disabled. It divided us very badly, because most of our issues need touch, others need to be held, others need to be directed by holding the hand.” – (Person with disability, Western Equatoria)

Of the households in Warrap and Western Equatoria who reported indirect effects of COVID-19, 29% and 23%, respectively, reported COVID-19 having an effect on their ability to access or utilise care [Table 3] while in Central Equatoria only 5% of households perceived such indirect effects. The main reason households reported being dissuaded from seeking care was because similar symptoms could be mistaken for COVID-19, whereas a smaller proportion of households reported a lack or the cancellation of health services, or the inability to visit health providers.

#### Community response to COVID-19

A discrepancy in community responses to COVID-19 was observed between the interview and household survey data., both in terms of communities’ general response towards the pandemic as well as pandemic-induced changes in health-seeking behaviour.

Many participants working in the health sector felt that communities were generally adhering well to the governmental COVID-19 guidelines, whereas other participants felt the exact opposite. Among the latter, health sector workers either perceived communities were ignorant of the guidelines or unwilling to follow them, or unable to do so as communities could not afford or access the necessary protective equipment. In contrast, the household survey data revealed the majority (92%) of households reported to have taken precautions to protect themselves against COVID-19 [Supplementary Table 2, Additional File 1], ranging from 73% of households in Warrap to 96% in Central Equatoria and 99% in Western Equatoria (chi^2^ (6) = 191.5978, *P* = 0.0000). Of these households, almost all reported to have taken two or more precautions, mainly hand-washing and disinfection of objects (76%), staying at home (65%), social distancing, reduced visiting of crowded areas (46%), and wearing face-masks (38%).

Some interview participants reported pandemic-induced changes in health seeking behaviour, which they ascribed to communities’ fears of visiting health facilities for COVID-19-related reasons. These included the fear of stigmatisation from the community by being seen at a health facility, or the fear of getting infected with COVID-19 at the health facility. Another commonly mentioned fear, was that of immediate diagnosis with COVID-19 without proper examination:

“There was something that happened to a certain guy who had asthma, so he was taken to the hospital, the doctor immediately took the person in quarantine without injecting the drug for asthma, until the person [died and was brought home]. The health workers should first identify the diseases” – (Adult community member, Central Equatoria)

However, among the household survey respondents who had a perceived need for care but chose not to seek it, less than one percent cited COVID-19-related reasons as their motivating factor [Table 3]. This was consistent with answers given by individuals who sought care at a provider that was not their closest [Supplementary Table 3, Additional File 1]. Similarly, most interview participants from communities reported that COVID-19 did not dissuade them from seeking care. Twenty-two participants working in the health sector reported communities stopped or delayed seeking care due to COVID-19, whereas 19 health sector participants mentioned communities were still visiting hospitals and health facilities as they used to do pre-pandemic.

The proportion of survey respondents reporting to have sought care at a health facility for a COVID-19 test was negligible (less than 1%) [Table 3], yet some respondents did report using community health worker services for COVID-19: 7% of community health worker services provided to households was related to COVID-19 services or information. This was mainly observed in Central Equatoria (26%) and, to a lesser extent, in Warrap (15%), whereas the proportion of households from Western Equatoria reporting to have used community health worker services for COVID-19 was negligible (1%) (chi^2^ (2) = 39.5134, *P* = 0.0000). Participants working in the health sector mentioned the training of Boma and community health workers was an important part of the COVID-19 awareness response, and several participants recognised the importance of community health workers raising awareness about COVID-19 at the community level. Some participants expressed doubts about the effectiveness of community health workers’ work for COVID-19.

“You find the Boma health workers they just… they are just there, they do not… they come, they greet like… Like the awareness is not strong when it comes to COVID-19, they live normally, as if there is no COVID-19” (HPF employee, Western Equatoria)

This lack of awareness was mainly ascribed to insufficient training of Boma and community health workers. Even though several participants mentioned these trainings were arranged often and were effective, some participants expressed they felt insufficiently trained: even though they were aware of COVID-19, they did not feel knowledgeable enough to make their patients aware of the risks and preventive measures associated with COVID-19.

The integrated findings from the qualitative and quantitative strands are visualised in Table 4.

**Table 4:** integrated findings from the qualitative and quantitative strands

Theme	Quantitative data	Qualitative data	Example quotation
*Community knowledge about COVID-19*			
Source of knowledge	Households mentioned multiple sources of health-related information (supplementary table 4, additional file 1) across the three states	Almost all participants mentioned to have heard of COVID-19, through different channels	*“I heard about the disease of COVID-19 over the radio” (Adolescent community member, Western Equatoria)* *“When I heard about Corona I was in the church.” (Adolescent Community member, Warrap)* *“In the market and everywhere else. l hear a lot of things being said about Coronavirus.” (Adolescent community member, Central Equatoria)*
Awareness of COVID-19 symptoms	n/a	Participants were well-aware of COVID-19 symptoms	*"It is a dangerous disease, it kills, people sneeze, it gives people difficulties in breathing, general rise of body temperature” (Person living with disability, Warrap)* *“If it has affected you, you feel body weakness, you cannot breathe very well, headache” (Adult community member, Central Equatoria)*
Awareness of COVID-19 preventive measures	n/a	Participants were well-aware of COVID-19 preventive measures	*“Then if it happens, you have to call the health worker, then call the number 666, then we have to protect ourselves by wearing the masks, then the hand sanitizers, washing” (Adult community member, Central Equatoria)* *“Protection is needed, you must wash your hands clean, do not shake hands, and so you see a fellow and just wave to them” (Adolescent community member, Western Equatoria)* *“People have to sit separate; one can sit there and another person there” (Adult community member, Warrap)*
*Perceived impact of COVID-19 on communities’ livelihoods*			
Direct health	Of the total number of individuals seeking care for a perceived need (*N* = 3,286), a negligible proportion did so to get tested for coronavirus	Not reported by interview participants	*n/a*
Non-health effects	In total, 79% of households (*N* = 1,223) perceived COVID-19 had affected their livelihoods in other ways than getting infected with the virus	Participants reported COVID-19 led to a lack of education due to school closures, teenage pregnancies, a lack of economic activities and church closures	*“This Coronavirus has come to spoil all our things. There is nothing good, young men have resorted to doing useless things” (Adult community member, Western Equatoria)* *“During the COVID-19, the people were not happy because it made life difficult” (Health facility worker, Western Equatoria)* *‘It closed schools and markets; markets used to function but they wanted to close markets, it has been a great challenge to people” (Adult Community member, Western Equatoria)*
Indirect health effects	Of the households in Warrap and Western Equatoria who reported indirect effects of COVID-19, 29% and 23%, respectively, reported an effect of COVID-19 on their ability to access or utilise care [Table 2] while in Central Equatoria only 5% of households perceived such indirect effects	Some interview participants working in the South Sudanese health sector reported COVID-19 resulted in the reduced ability to access health services for local populations, due to reduced opening hours of health facilities, reduced availability of essential medicines and equipment, or reduced quality of care at facilities	*“With the coming of coronavirus people [at the health facility] are working from Monday to Friday only and is not on full time, at 1:00 pm people have already left for home. Before that, people used to work from 7:00 am to 5:00 pm from Monday to Saturday” (Boma Health Committee member, Central Equatoria)* *“The issue of Corona has scared people; it does not allow people to stay close to each other, it has created distancing. But that is not why people stopped going to the hospital, only that hospitals are not there” (Traditional Leader, Warrap)*
*Community response to COVID-19*			
Adherence to governmental COVID-19 guidelines	The majority (92%, *N* = 1,223) of households reported to have taken precautions to protect themselves against COVID-19	Many participants working in the health sector felt that communities were generally adhering well to the governmental COVID-19 guidelines, whereas other participants felt the exact opposite	*"I do not see them wearing mask, and people in the market are not wearing masks” (NGO employee, Western Equatoria)* *“When we raise awareness, they do not accept what we are talking about, like actually COVID-19 is affecting many people, and it is the ignorance of the rules and regulations that are in the Ministry of Health” (Local Media employee, Warrap)* *“For this COVID-19 from the beginning the community, when they come to the facility, we tell them to wear the mask, wash their hands. They did not want, but after health education, they are now wearing face mask, washing hands and measuring their temperature.” (Health Facility Worker, Central Equatoria)*

## Discussion

The aim of this study was to understand the effect of COVID-19 on healthcare access and South Sudan’s healthcare response from the onset of the COVID-19 pandemic until September 2021. Before reflecting on South Sudan’s COVID-19 response, we will first reinterpret the findings related to COVID-19’s impact on healthcare access and utilisation within existing literature and similar studies.

In line with observations in other Sub-Saharan African countries, official COVID-19 surveillance in South Sudan shows a limited number of infections and low mortality rates, contrary to what was seen in other continents and contrary to projections made at the beginning of the pandemic [45, 46]. In an analysis conducted by Bamgboye et al. [45], limited testing capacity in African countries was one of the major reasons explaining this difference. An assessment by the WHO conducted in late 2021 showed that only one in seven (14.2%) COVID-19 infections was detected in Africa [47]. With a total of approximately 340,000 COVID-19 tests conducted on a population of 11 million by March 2021, South Sudan lagged behind other fragile and conflict-affected settings of similar population size in terms of number of tests conducted: by March 2021, Burundi had conducted over 1.4 million COVID-19 tests and Rwanda was close to conducting a total of 5 million COVID-19 tests [44, 48]. According to latest reports from the COVID-19 Data Repository of John Hopkins University, the positivity rate in South Sudan has skyrocketed during both South Sudan’s COVID-19 waves (early 2020 and early 2021), with up to 20%-35% of tests confirming a COVID-19 case [3, 44]. Since the WHO considers a positivity rate of less than 10% a benchmark for adequate testing, it seems likely that testing capacity in the country was not able to capture the magnitude of COVID-19 surges in South Sudan.

Nevertheless, our results suggest that the magnitude and severity of transmission have remained relatively moderate during the first 1.5 years of COVID-19 in South Sudan, especially outside of Juba. However, our measurement of this was based on self-reports: no interview participants reported to have been infected with COVID-19 themselves, or know anyone who knowingly had, and the proportion of survey respondents having sought care for a COVID-19 test was negligible. Indirect effects of COVID-19, both on health seeking as on livelihoods in general, were reported by some household survey respondents, but direct health effects of COVID-19 were not observed. Both observations from health system managers and recently published newspaper articles do not indicate actual numbers of infections have greatly exceeded official surveillance.

Even so, limited empirical surveillance of COVID-19 transmission hampers South Sudan’s COVID-19 response and management [49] by both affecting the ability to gauge the problem COVID-19 poses to the country, as well as the ability to balance a COVID-19 response alongside the country’s other pressing epidemiological priorities and routine health service delivery. Improved surveillance of COVID-19 transmission is of immediate priority.

The fact that South Sudan’s testing capacity for COVID-19 remains severely limited to date makes it difficult to draw definitive conclusions on the impact of the pandemic on any aspect of communities' lives, including access to healthcare. However, our results suggest that, even though some fears related to COVID-19 may have been present among communities, these have not greatly dissuaded people from seeking care and do not yield significant consequences for health system programming in South Sudan. For example, some households in Western Equatoria did report an effect of COVID-19 on their health seeking, but this was mainly that similar symptoms would be mistaken for COVID-19. Given our finding that awareness among communities of COVID-19 was good, the country could not have done much more to motivate appropriate health seeking behaviour in this case. In Central Equatoria, perceived indirect effects of COVID-19 were not often related to health seeking, and the majority of effects observed, in Central and Western Equatoria at least, were on people’s livelihoods, most notably a loss of income or disrupted education. Major variations exist in Sub-Saharan Africa in terms of COVID-19's impact on healthcare access, confounded with the impact of lockdown measures [14–16], reflecting the generally wide variation in healthcare access and utilisation on the continent. However, a recent analysis of COVID-19’s impact on service utilisation rates in the DRC, a country with extremely-low health services utilisation rates, found an immediate decrease in the utilisation of health services following the onset of the COVID-19 pandemic in Kinshasa [17, 50]. Furthermore, the decline in health service utilisation was found to be mostly due to the lockdown policy, whereas the pandemic itself was less of an influencing factor. The fact that our study shows contrasting findings may be due to several factors, such as the difference in COVID-19 epidemiology in both countries, the different timing of both studies and the variations in urban–rural context. Another contributing factor may be that our findings are centred around self-reported perceived changes in healthcare access and utilisation, as opposed to an analysis of health service utilisation data in the case of DRC [17].

Despite COVID-19’s limited number of officially reported infections and low mortality rates in South Sudan, continued transmission through asymptomatic patients yields a bigger question for global COVID-19 response efforts, as continued circulation of the virus has the potential to generate new variants [51]. An important question is therefore whether efforts should be made to curb this transmission in South Sudan, such as through an improved COVID-19 vaccine roll-out. Such efforts may conflict with other local health priorities, such as malaria, and considering that we did not observe a major impact of COVID-19 on health seeking behaviours. Furthermore, our findings also suggest that a health shock such as COVID-19 caused a greater reliance on, and increased responsibility for, international donors for health planning. This aligns to observations from the tenth EVD outbreak in the DRC, in which the provision of technical assistance from international organisations during the emergency was observed to build a parallel and short-time outbreak response rather than providing the much-needed reinforcement of the generally-weak healthcare sector [52]. Given the fact that priorities on COVID-19 are greatly shifting (i.e., mandatory vaccination, removal of travel bans), these dynamics may be useful to consider in further COVID-19 planning, particularly regarding any vaccine roll-out. South Sudan must now navigate a period of transition where COVID-19 vaccine roll-out continues while other domestic priorities are re-strengthened.

### Strengths & limitations

To our knowledge, our study was the first to assess the impact of COVID-19 on healthcare access and utilisation in South Sudan. As far as we know, this is also the first comprehensive mixed methods study into healthcare access and health seeking behaviour in South Sudan. The inclusion of three states with different social, economic, cultural and political realities means the populations we drew our sample from represent a diversity of experiences in healthcare needs, access, utilisation and quality. Data collection was conducted by a team of locally-knowledgeable and competent researchers and enumerators, enabling interviews and surveys to be conducted in appropriate languages for each state, and transcripts were immediately translated to English for collation and analysis. This knowledge was invaluable for revising our research tools so questions were relevant, appropriate and could be readily understood by all data collection team members and research participants. We also implemented a thorough quality assurance strategy to detect and correct errors or logical inconsistencies in data, which heightened its quality. Nevertheless, while interviews and FGDs were conducted in five different languages across states (namely Juba Arabic, Arabic, Dinka, Bari and Azande), there were still instances where participants had to speak languages other than their mother tongue, due to the great linguistic variation in the country. This will have impeded their understanding of some questions and their prerogative to be understood. Another limitation that needs to be recognized is that this study focuses on the first 1.5 years of the COVID-19 pandemic only. Changes in COVID-19’s epidemiology and vaccine roll-out since July 2021 may result in some findings no longer be accurate at the time of publication [3]. Further, despite our efforts to limit the effects of social desirability bias, fears and stigmatisation regarding COVID-19 may be underreported. Last, we used data collected in three of South Sudan’s twelve states, meaning certain state-level dynamics may not be captured. Nonetheless, considering the three states were purposively chosen to represent three distinct South Sudanese settings, the results are believed to provide an accurate representation of the situation across much of South Sudan.

### Recommendations

Given the evolving nature of the pandemic and its impact on health systems and society, it is important to consider what lessons can be drawn from this study for both future research and the health systems response to the pandemic. First, beyond focusing on indicators such as the supply of reagents and qualified laboratory staff, improving diagnostic capacity in an effective and sustainable manner also requires the alignment of donor targets and funding to ensure inputs can lead to testing readiness [53]. The Organisation for Economic Co-Operation and Development’s framework for Donor Co-Operation [54] could be particularly useful to achieve this. In terms of setting targets, moving away from the provision of inputs to effective testing capacity can be helped by setting output-, outcome- and impact-based targets, such as on the timely diagnosis and treatment of COVID-19. Additionally, surveillance and response strategies should be informed by operational research to areas of public health importance in order to ground them in local settings [53, 55].

Second, to supplement official COVID-19 surveillance, point-in-time questionnaires, patient interviews or other verbal reporting mechanisms may help improve surveillance of COVID-19 transmission in contexts where testing or surveillance is limited, particularly in countries where the national government is coping with significant resource and capacity constraints, such as in South Sudan [56, 57]. Assessing relatively rare cases of mortality which may carry stigma through survey instruments in a fragile or conflict-affected context, however carries limitations as well, often leading to underreporting, as found in relation to measurement of maternal mortality in Afghanistan [58]. More research is therefore needed into the way verbal reporting mechanisms can aid in assessing the true public health impact, including mortality, of current and future disease outbreaks [56].

Third, the insights from this study, based on self-reporting, are best combined with an analysis of actual healthcare utilisation trends in South Sudan. This can also help inform health planners and programmers where support is most needed. Since we found individuals perceived the effect of the pandemic to be mostly on non-health-related aspects of their lives, the support of basic needs, such as food, could be helpful in protecting South Sudanese people from further socioeconomic hardship, particularly those most vulnerable. Additionally, it would be valuable to consider communities’ prioritisation of the various health problems present in the South Sudanese context, outbreaks and chronic health challenges alike, particularly as this should inform the Ministry of Health’s own prioritisation of resources. In line with other literature on the perceived changes to healthcare access during shocks, we recommend to further explore how perceptions about COVID-19 actually change health seeking behaviours, as well as the effect of factors such as mobility restrictions and public information campaigns on these health perceptions and preferences.

## Conclusion

We found that fears related to COVID-19 have not greatly dissuaded people from seeking care and that these did not yield significant consequences for health system programming in South Sudan. In fact, the majority of COVID-19 effects observed were on people’s livelihoods, most notably loss of income and disrupted education. We also found that implementation of COVID-19 response activities sometimes created frictions between the national government and international health actors in South Sudan. Given the fact that global priorities on COVID-19 are greatly shifting, these dynamics must be considered in further COVID-19 planning, particularly the vaccine roll-out. South Sudan must now navigate a period of transition where COVID-19 vaccine roll out continues while other domestic health needs are re-prioritised.

## Supplementary Information

**Additional file 1: Supplementary table 1.** perceived effect of COVID-19 on household’s livelihood by wealth index quintile (right most part of the table continued below). **Supplementary table 2.** households having taken precautions to protect themselves against COVID-19, stratified by state. **Supplementary table 3.** reasons for not visiting closest provider. **Supplementary table 4.** sources of health information, including information on COVID-19, reported by households.

## Data Availability

Qualitative data are not publicly available to protect privacy of the participants, but are available on reasonable request on a case-by-case basis from the primary author M.A.S. Schots.

## Abbreviations

AIDS: Acquired immuno deficiency syndrome
BHI: Boma Health Initiative
COVID-19: Coronavirus Disease 2019
COVAX: COVID-19 Vaccines Global Access
DRC: Democratic Republic of the Congo
EVD: Ebola Virus Disease
FGD: Focus Group Discussion
HIV: Human immunodeficiency virus
HPF: Health Pooled Fund
HPF3: Health Pooled Fund Phase 3
KIT: KIT Royal Tropical Institute
NGO: Non-Governmental Organisation
ODK: Open Data Kit
PSU: Primary sampling unit
WHO: World Health Organization

## Footnotes

1 including the British Government’s Foreign, Commonwealth and Development Office, Global Affairs Canada, the European Union, the Swedish International Development and Cooperation Agency, the United States Agency for International Development, and Gavi, the Vaccine Alliance.
