# Supplementary material for: The impact of the COVID-19 pandemic on healthcare access and utilisation in South Sudan: a cross-sectional mixed methods study
Source: BMC Health Serv Res. 2022 Dec 20;22:1559. doi: 10.1186/s12913-022-08929-9 (PMC9765347; doi:10.1186/s12913-022-08929-9)
Supplement: Supplementary file 1 — Additional file 1: Supplementary table 1. perceived effect of COVID-19 on household’s livelihood by wealth index quintile (right most part of the table continued below). Supplementary table 2. households having taken precautions to protect themselves against COVID-19, stratified by state. Supplementary table 3. reasons for not visiting closest provider. Supplementary table 4. sources of health information, including information on COVID-19, reported by households. [file 12913_2022_8929_MOESM1_ESM.docx]

## Additional file 1

Supplementary table 1: perceived effect of COVID-19 on household’s livelihood by wealth index quintile (right most part of the table continued below).

| **Indicator** | **Lowest** | | | **Second** | | | | **Middle** | | | |  |
| --- | --- | --- | --- | --- | --- | --- | --- | --- | --- | --- | --- | --- |
|  | **N** | **%** | **SE** | | **N** | **%** | **SE** | | **N** | **%** | **SE** | |
| **Households total** | 305.5 |  |  | | 287.9 |  |  | | 284.8 |  |  | |
| **Households reporting indirect effect of COVID-19** | 221 | 72.5 | 3.9 | | 215 | 74.7 | 2.8 | | 221 | 77.8 | 3.4 | |
| **Indirect effect on health seeking** |  |  |  | |  |  |  | |  |  |  | |
| *Mistaken similar symptoms for COVID-19* | 32 | 14.4 | 5.2 | | 24 | 11.1 | 3.6 | | 23 | 10.2 | 2.3 | |
| Lack/cancellation of other health services | 6 | 2.6 | 1.2 | | 20 | 9.2 | 2.7 | | 10 | 4.6 | 1.8 | |
| Other health problems due to not visiting a health provider | 9 | 4 | 1.4 | | 22 | 10.4 | 3.2 | | 11 | 5.1 | 2.2 | |

(supplementary table 1 continued)

| **Indicator** | **Fourth** | | | **Highest** | | | | **Total** | | | |  |
| --- | --- | --- | --- | --- | --- | --- | --- | --- | --- | --- | --- | --- |
|  | **N** | **%** | **SE** | | **N** | **%** | **SE** | | **N** | **%** | **SE** | |
| **Households total** | 198.4 |  |  | | 146.5 |  |  | | 1,223 |  |  | |
| **Households reporting indirect effect of COVID-19** | 172 | 86.5 | 2.3 | | 135 | 91.8 | 1.7 | | 964 | 78.8 | 1.8 | |
| **Indirect effect on health seeking** |  |  |  | |  |  |  | |  |  |  | |
| *Mistaken similar symptoms for COVID-19* | 12 | 6.9 | 2.3 | | 2 | 1.6 | 1.3 | | 92 | 9.6 | 1.5 | |
| Lack/cancellation of other health services | 2 | 1.4 | 0.8 | | 1 | 0.5 | 0.5 | | 39 | 4 | 0.6 | |
| Other health problems due to not visiting a health provider | 8 | 4.9 | 2.0 | | 2 | 1.7 | 0.9 | | 53 | 5.5 | 1.2 | |

Supplementary table 2: households having taken precautions to protect themselves against COVID-19, stratified by state.

| **Indicator** | **Central Equatoria** | | | **Warrap** | | | **Western Equatoria** | | | **Total** | | |
| --- | --- | --- | --- | --- | --- | --- | --- | --- | --- | --- | --- | --- |
|  | **N** | **%** | **SE** | **N** | **%** | **SE** | **N** | **%** | **SE** | **N** | **%** | **SE** |
| **Households total** | 224 |  |  | 290 |  |  | 709 |  |  | 1,223 |  |  |
| **Households taken at least one precaution for COVID-19** | 216 | 96.3 | 2.2 | 212 | 73.1 | 2.2 | 699 | 98.6 | 0.5 | 1,127 | 92.1 | 0.9 |
| *Precautions taken to protect against COVID-19* |  |  |  |  |  |  |  |  |  |  |  |  |
| Tried to stay home as much as possible | 93 | 43.2 | 2.3 | 84 | 39.8 | 2.9 | 556 | 79.6 | 3.3 | 734 | 65.1 | 1.9 |
| Prayer | 12 | 5.4 | 1.3 | 24 | 11.5 | 2.1 | 182 | 26 | 2.4 | 218 | 19.3 | 1.8 |
| Social distancing | 137 | 63.4 | 3.7 | 131 | 61.8 | 3.9 | 248 | 35.5 | 2.8 | 516 | 45.8 | 2.5 |
| Reduced travel in general | 35 | 16 | 2.4 | 23 | 10.8 | 2.1 | 104 | 14.9 | 1.7 | 161 | 14.3 | 1.2 |
| Wearing masks | 111 | 51.4 | 3.6 | 99 | 46.7 | 3.3 | 221 | 31.7 | 2.9 | 431 | 38.3 | 2.0 |
| Handwashing and disinfection of objects | 189 | 87.4 | 2.1 | 159 | 75.1 | 3.4 | 504 | 72.2 | 3.1 | 852 | 75.6 | 2.2 |
| Avoided health facilities out of fear of infection | 3 | 1.4 | 0.7 | 6 | 2.8 | 1.0 | 13 | 1.8 | 0.6 | 22 | 1.9 | 0.5 |
| Other | 6 | 2.6 | 0.9 | 3 | 1.3 | 0.6 | 0 | 0 | 0 | 8 | 0.7 | 0.2 |

Supplementary table 3: reasons for not visiting closest provider

| **Indicator** | **Central Equatoria** | | | **Warrap** | | | **Western Equatoria** | | | **Total** | | |
| --- | --- | --- | --- | --- | --- | --- | --- | --- | --- | --- | --- | --- |
|  | **N** | **%** | **SE** | **N** | **%** | **SE** | **N** | **%** | **SE** | **N** | **%** | **SE** |
| **Individuals with a perceived need for care** | 767 | 49.7 | 1.8 | 996 | 52.6 | 1.8 | 2,142 | 47.8 | 1.9 | 3,905 | 49.3 | 1.1 |
| **Individuals not seeking care at the closest health provider** | 125 | 22.5 | 2.4 | 177 | 29.9 | 2.7 | 127 | 6.6 | 1.5 | 429 | 13.9 | 1.6 |
| **At least one reason for not visiting the closest health provider** | 124 | 98.2 | 1.0 | 174 | 98.2 | 1.0 | 127 | 100 | 0 | 425 | 99.1 | 0.4 |
| *Reasons for not visiting the closest health provider* |  |  |  |  |  |  |  |  |  |  |  |  |
| Related to COVID-19 | 0 | 0 | 0 | 1 | 0.6 | 0.4 | 0 | 0 | 0 | 1 | 0.2 | 0.2 |
| Not related to COVID-19 | 237 | 189.2 | 36.7 | 370 | 208.3 | 40.6 | 338 | 265.5 | 78.1 | 943 | 219.7 | 34.4 |

Supplementary table 4: sources of health information, including information on COVID-19, reported by households

| **Indicator** | **Central Equatoria** | | | **Warrap** | | | **Western Equatoria** | | | **Total** | | |
| --- | --- | --- | --- | --- | --- | --- | --- | --- | --- | --- | --- | --- |
|  | **N** | **%** | **SE** | **N** | **%** | **SE** | **N** | **%** | **SE** | **N** | **%** | **SE** |
| **Households total** | 224 |  |  | 290 |  |  | 709 |  |  | 1,223 |  |  |
| **Households haven used 2 or more sources of health information** | 191 | 85.3 | 0 | 226 | 77.9 | 0 | 576 | 81.3 | 0 | 993 | 81.2 | 0 |
| *Sources of health information* |  |  |  |  |  |  |  |  |  |  |  |  |
| Relatives/friends | 68 | 30.4 | 1.6 | 167 | 57.7 | 4.9 | 233 | 32.9 | 1.7 | 469 | 38.3 | 1.7 |
| Community leaders | 101 | 44.8 | 2.0 | 74 | 25.7 | 2.3 | 230 | 32.4 | 2.7 | 405 | 33.1 | 1.8 |
| Health workers at facilities or clinics | 33 | 14.6 | § | 71 | 24.4 | 1.8 | 38 | 5.4 | 1.4 | 142 | 11.6 | 1.3 |
| Church | 42 | 18.9 | 3.0 | 29 | 10.2 | 2.3 | 508 | 71.7 | 2.8 | 580 | 47.4 | 1.6 |
| Boma/Community health workers | 16 | 7.3 | 2.1 | 11 | 3.7 | 1.0 | 71 | 10.0 | 1.6 | 98 | 8.0 | 1.1 |
| Boma Health Committee | 5 | 2.2 | 0.8 | 17 | 5.8 | 1.3 | 32 | 4.4 | 1.3 | 53 | 4.4 | 0.8 |
| County health team | 9 | 4.2 | 0.9 | 10 | 3.3 | 1.1 | 7 | 1.0 | 0.6 | 26 | 2.2 | 0.5 |
| EPI outreach teams | 19 | 8.3 | 1.8 | 6 | 1.9 | 1.0 | 0 | 0 | 0.0 | 24 | 2.0 | 0.5 |
| Religious/traditional healers | 0 | 0 | 0.0 | 3 | 1.2 | 0.6 | 2 | 0.3 | 0.3 | 6 | 0.5 | 0.2 |
| Radio | 129 | 57.5 | 2.9 | 135 | 46.5 | 4.7 | 347 | 49 | 3.9 | 611 | 50.5 | 2.5 |
| TV | 13 | 5.9 | 2.2 | 1 | 0.3 | 0.3 | 9 | 1.3 | 0.9 | 23 | 1.9 | 0.6 |
| Internet | 2 | 0.7 | 0.4 | 1 | 0.4 | 0.3 | 0 | 0.0 | 0.0 | 3 | 0.2 | 0.1 |
| Social media | 6 | 2.7 | 1.1 | 2 | 0.6 | 0.4 | 0 | 0.0 | 0.0 | 8 | 0.6 | 0.2 |
| Posters/pamphlets/other literature | 1 | 0.6 | 0.4 | 0 | 0.0 | 0.0 | 0 | 0.0 | 0.0 | 1 | 0.1 | 0.1 |
| On the road | 9 | 4.2 | 0.9 | 20 | 6.8 | 1.7 | 4 | 0.6 | 0.3 | 33 | 2.7 | 0.5 |
| megaphone | 102 | 45.7 | 4.0 | 66 | 22.8 | 3.3 | 119 | 16.7 | 3.2 | 287 | 23.5 | 2.5 |
